# Supplementary figures and images for: Spiny mice (Acomys) exhibit attenuated hallmarks of aging and rapid cell turnover after UV exposure in the skin epidermis
Source: PLoS One. 2020 Oct 30;15(10):e0241617. doi: 10.1371/journal.pone.0241617 (PMC7598470; doi:10.1371/journal.pone.0241617)

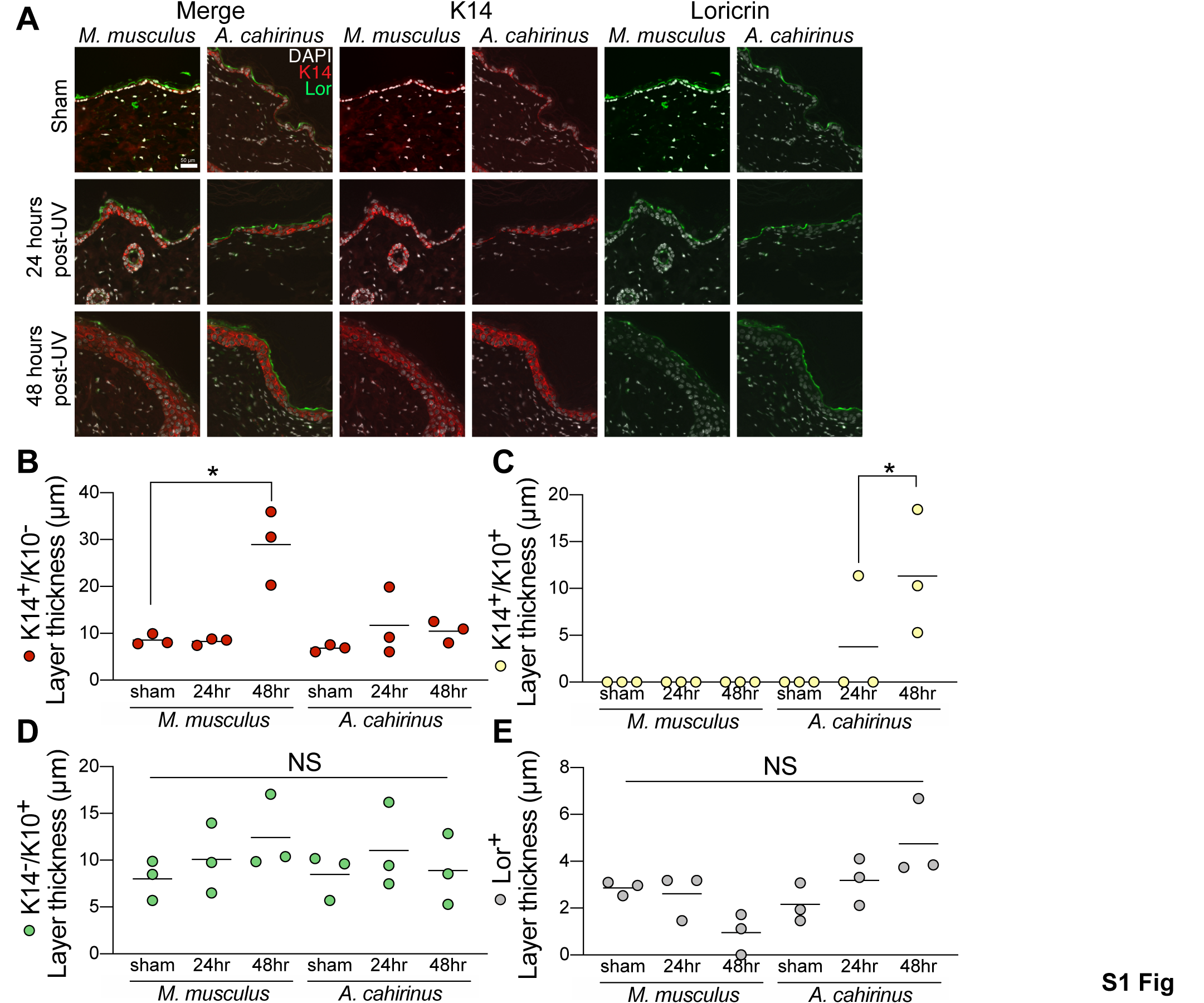

Supplement: S1 Fig — A, representative immunofluorescence images of epidermal differentiation markers keratin 14 (K14) and loricrin (Lor) labeling. B, C, D, E individual layer thickness quantification of the (B), K14+/K10– single positive basal layer (C), K14+/K10+ double positive middle suprabasal layer (D), K14–/K10– single positive spinous layer and (E), Lor+ single positive cornified envelope. n = 3 animals per group. Data points are biological replicates and lines indicate group means. *Significantly different (p<0.05) from the indicated group. (TIF) [file pone.0241617.s001.tif]

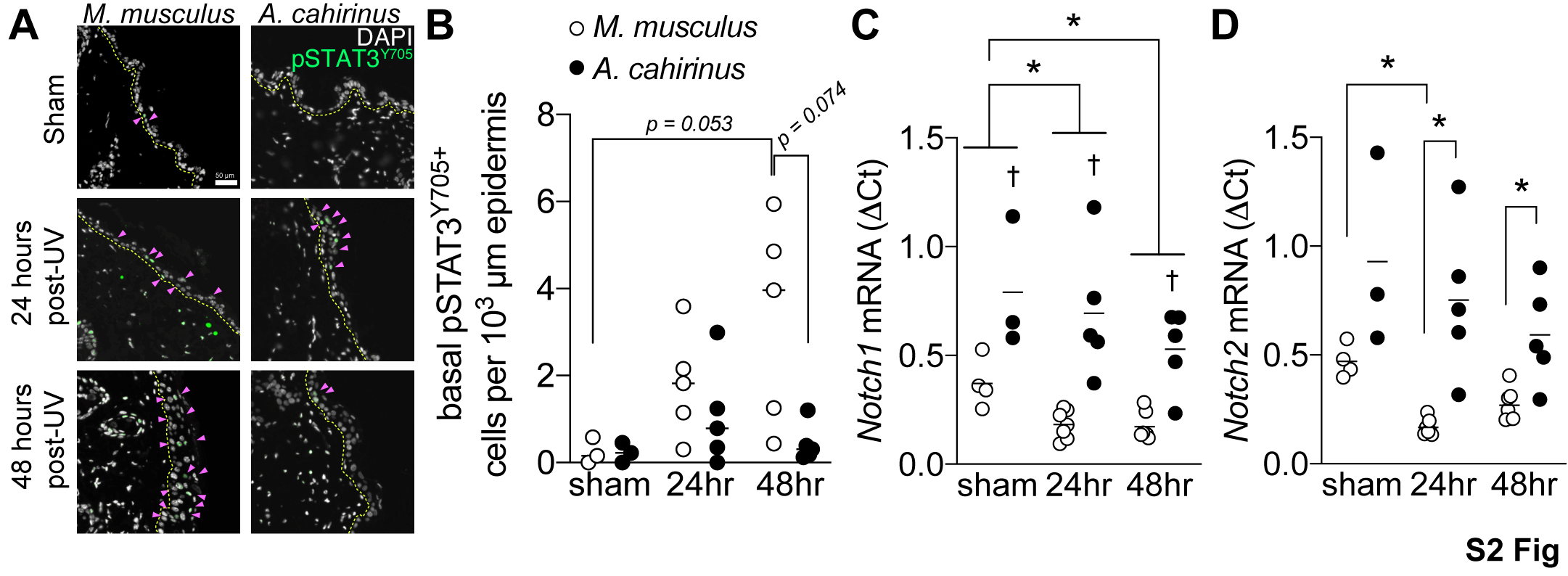

Supplement: S2 Fig — A, representative immunofluorescence images of epidermal nuclear phosphorylated STAT3 on Y705 (pSTAT3Y705) labeling of skin from control (sham) and UV-irradiated M. musculus and A. cahirinus, collected 24 and 48 hours after exposure. Positive cells are indicated by the pink arrows and the epidermal basement membrane is indicated by the yellow dashed line. Scale bar = 50 μm. B, quantification of pSTAT3Y705 labeling in basal epidermis. n = 3 sham of each species; n = 5 animals at 24hr and 48hr from each species. C, Notch1 and D, Notch2 mRNA expression in whole skin from UV-irradiated M. musculus and A. cahirinus, collected from sham controls or 24 and 48 hours after UVB exposure. For all qPCR: sham, n = 3–4 each species; 24hr n = 5–7 each species; 48hr M. musculus, n = 5–6; 48hr A. cahirinus, n = 3–5 animals per group. All data points are biological replicates and lines indicate group means. *Significantly different (p<0.05) from the indicated group. †Significant (p<0.05) overall effect of species. (TIF) [file pone.0241617.s002.tif]
